# Identification of Immunological Parameters as Predictive Biomarkers of Relapse in Patients with Chronic Myeloid Leukemia on Treatment-Free Remission

**DOI:** 10.3390/jcm10010042

**Published:** 2020-12-25

**Authors:** Lorena Vigón, Alejandro Luna, Miguel Galán, Sara Rodríguez-Mora, Daniel Fuertes, Elena Mateos, Miguel Piris-Villaespesa, Guiomar Bautista, Esther San José, José Rivera-Torres, Juan Luis Steegmann, Fernando de Ory, Mayte Pérez-Olmeda, José Alcamí, Vicente Planelles, María Rosa López-Huertas, Valentín García-Gutiérrez, Mayte Coiras

**Affiliations:** 1 Immunopathology Unit, National Center of Microbiology, Instituto de Salud Carlos III, 28220 Madrid, Spain; lvhernandez@isciii.es (L.V.); srmora@isciii.es (S.R.-M.); emateo@isciii.es (E.M.); ppalcami@isciii.es (J.A.); mrlhuertas@isciii.es (M.R.L.-H.); 2 Hematology Service, Instituto Ramón y Cajal de Investigación Sanitaria (IRYCIS), Hospital Universitario Ramón y Cajal, 28034 Madrid, Spain; lunadeabia@gmail.com (A.L.); mpirisv@gmail.com (M.P.-V.); 3 Pharmacy and Biotechnology Department, Faculty of Biomedical Sciences, Universidad Europea de Madrid, 28670 Madrid, Spain; miguel-galan-burgos@hotmail.com (M.G.); ESTHER.SANJOSE@universidadeuropea.es (E.S.J.); JOSE.RIVERA@universidadeuropea.es (J.R.-T.); 4 School of Telecommunications Engineering, Universidad Politécnica de Madrid, 28040 Madrid, Spain; danifuertes100@gmail.com; 5 Hematology Service, Hospital Universitario Puerta de Hierro, 28222 Madrid, Spain; guiomarbautista@gmail.com; 6 Hematology Service, Hospital Universitario La Princesa, 28006 Madrid, Spain; jlsteegmann@gmail.com; 7 Serology Service, National Center of Microbiology, Instituto de Salud Carlos III, 28220 Madrid, Spain; ernandodeorym@gmail.com (F.d.O.); MAYTEPEREZ@isciii.es (M.P.-O.); 8 Division of Microbiology and Immunology, University of Utah School of Medicine, Salt Lake City, UT 84132, USA; vicente.planelles@path.utah.edu

**Keywords:** chronic myeloid leukemia, treatment-free remission, immunological response

## Abstract

BCR-ABL is an aberrant tyrosine kinase responsible for chronic myeloid leukemia (CML). Tyrosine kinase inhibitors (TKIs) induce a potent antileukemic response mostly based on the inhibition of BCR-ABL, but they also increase the activity of Natural Killer (NK) and CD8+ T cells. After several years, patients may interrupt treatment due to sustained, deep molecular response. By unknown reasons, half of the patients relapse during treatment interruption, whereas others maintain a potent control of the residual leukemic cells for several years. In this study, several immunological parameters related to sustained antileukemic control were analyzed. According to our results, the features more related to poor antileukemic control were as follows: low levels of cytotoxic cells such as NK, (Natural Killer T) NKT and CD8±TCRγβ+ T cells; low expression of activating receptors on the surface of NK and NKT cells; impaired synthesis of proinflammatory cytokines or proteases from NK cells; and HLA-E*0103 homozygosis and KIR haplotype BX. A Random Forest algorithm predicted 90% of the accuracy for the classification of CML patients in groups of relapse or non-relapse according to these parameters. Consequently, these features may be useful as biomarkers predictive of CML relapse in patients that are candidates to initiate treatment discontinuation.

## 1. Introduction

Chronic myeloid leukemia (CML) is a clonal hematopoietic stem cell (HSC) disorder characterized by the presence of Philadelphia (Ph) chromosome, which is produced by a reciprocal translocation between chromosomes 9 and 22, t(9;22)(q34;q11) [1]. This translocation gives rise to the BCR-ABL1 oncogene that is translated into a tyrosine kinase protein with constitutive activity, leading to the uncontrolled HSC proliferation [2]. CML has a natural course of 3 to 5 years and it roughly accounts for 15% of adult leukemia [3]. Therefore, CML is a major public health concern.

During treatment with tyrosine kinase inhibitors (TKIs), most patients with CML develop hematologic and cytogenetic responses and, after several years of treatment, they get a life quality comparable to that of the unaffected population [4,5]. Treatment interruption may be considered in patients who achieve a deep molecular response (DMR), which is defined as BCR-ABL1/ABL1 ratio ≤ 0.01%. When DMR is sustained for more than 2 years, patients are considered for an attempt at treatment-free remission (TFR). Although the great majority of patients who discontinue TKI treatment frequently develop DMR with undetectable levels of BCR-ABL1 transcript by qRT-PCR, only about half of the patients who discontinued treatment maintain TFR, whereas most patients relapse within 6 months after treatment interruption [6,7,8,9,10].

The duration of both TKI treatment and DMR have been associated with higher probability of sustained TFR [7,9], but other factors are also important, such as the patient’s clinical characteristics at diagnosis, the depth of early response to treatment, the mutations in BCR-ABL kinase domain [11] and most importantly, the control exerted by the immune system on the residual leukemic cells. In fact, it is improbable that all leukemic cells are completely eradicated during treatment and consequently, the residual leukemic load should be controlled after treatment interruption to avoid relapse [12]. In this regard, the digital PCR (dPCR) has emerged as a more sensitive and accurate technique than qRT-PCR for monitoring BCR-ABL1 transcript levels and, therefore, for predicting the patients more susceptible to relapse after discontinuation of TKI [13,14]. Therefore, the sustained control after treatment interruption should be a balance between the residual leukemic cells and the immune response. In fact, a leukemia-specific vaccine approach directed to the p210-derived breakpoint of BCR-ABL could almost eliminate the residual leukemic cells through specific CD4+ T cell responses [15]. Moreover, TKIs are immunomodulatory drugs that may induce a potent antileukemic response mostly based on the expansion of large granular lymphocytes (LGLs) with cytotoxic phenotype [16]. The main components of these LGLs, at least in patients treated with dasatinib, are Natural Killer (NK) cells [17,18]. However, not all patients may maintain this immunological control after treatment discontinuation and it is very difficult to predict which patients will relapse.

As the risk of relapse during TFR is highly dependent on the individual’s antileukemic response [12], the evaluation of the immune profile in blood cells from patients with CML that are about to discontinue treatment is essential to predict a sustained TFR [19]. Although CML patients on treatment with dasatinib develop increased levels of classical NK cells (CD3-CD56+) in comparison with other TKIs [20], long-term TFR is also feasible in patients treated with imatinib and nilotinib [6,21,22]. Therefore, it is necessary to determine which immunological parameters may better define the global response against leukemic cells after treatment with different TKIs in order to predict the outcome of TKI discontinuation. The cytotoxic activity of NK cells depends on the balance between inhibitory and activating signals that are generated from a wide variety of cell surface receptors such as CD94/NKG2 and the killer cell Ig-like receptors (KIRs) [23]. The levels of expression of some CD94/NKG2 receptors have been related to the antileukemic control during TFR, such as NKG2A downregulation [24] and NKG2C upregulation [25], which have been associated with reduced CML relapse during treatment interruption. Conversely, the presence of some KIR genes such as KIR2DS3 [26] and KIR2DL5B [27] have been related to CML relapse. Other cytotoxic cells may also be involved in the complex antileukemic response developed by TKIs, such as non-conventional CD3 + CD8 ± T lymphocytes expressing TCRγδ+ [28]. Similar to NK cells, TCRγδ+ lymphocytes may recognize cells with missing-self and are activated by antigens presented by HLA-E [29] or even without previous antigen processing [30]. Moreover, 70% of TCRγδ+ lymphocytes in peripheral blood express CD94/NKG2 and 10% express KIRs [31].

In summary, many clinical parameters may impact the outcome of CML after treatment interruption and several studies have tried to define biomarkers to predict successful TFR but results are contradictory so far [32,33,34]. Positive outcomes of treatment interruption have also been observed in patients who were on treatment with other immunomodulatory drugs such as IFNα, which highlights the importance of the immune response to control residual leukemic cells [7,35,36]. Consequently, in this study we analyzed several immunological parameters related to the antileukemic cytotoxic response in order to determine the best biomarkers to predict relapse of CML during controlled treatment interruption.

## 2. Experimental Methods

### 2.1. Participants

Blood samples were obtained from 93 patients with CML Ph+ in different stages of evolution (Table 1). Twenty healthy donors with similar age and gender distribution were recruited as controls. Peripheral blood mononuclear cells (PBMCs) were isolated by centrifugation through Ficoll-Hypaque gradient (Pharmacia Corporation, North Peapack, NJ, USA). Due to sample size limitations, not all the analyses were performed with all the samples.

### 2.2. Flow Cytometry Analysis

Antibodies for cell surface staining were CD3-APC, CD56-FITC, CD16-PercP, CD8-PercP (CD8α, clone SK1), KIR2DL5 (CD158f)-BV421, and NKG2D-PECy7 (BD Biosciences, San Jose, CA, USA). Antibody TCRγδ-PE was obtained from BioLegend (San Diego, CA, USA) and antibodies NKG2A-PE and NKG2C-AlexaFluor700 were obtained from R&D Systems (Minneapolis, MN, USA). CD8+ T cells were analyzed within the T lymphocyte gate. Both CD8+ and CD8-T cells were considered for the analysis of TCRγδ expression. CD56+ cells were analyzed within CD3 ± gate to differentiate between NK and NKT cells.

For intracellular staining of interferon gamma (IFNγ), tumor necrosis factor alfa (TNFα) and granzyme B (GZB) from NK cells, PBMCs were treated for 4h at 37 °C with Hsp70 peptide 1 μgr/mL (Abcam, Cambridge, UK) and brefeldin-A (BD Biosciences, San Jose, CA, USA). Cells were then stained with antibodies against CD3, CD56 and CD16. After fixation and permeabilization with IntraPrep Reagent (Beckman Coulter, Indianapolis, IN, USA), cells were stained with antibodies against IFNγ-PE (Beckman Coulter, Indianapolis, IN, USA), TNFα-PE (Beckman Coulter, Indianapolis, IN, USA) or GZB-PE (BD Biosciences).

Data acquisition was performed in a BD LSRFortessa X-20 flow cytometer using FACS Diva software (BD Biosciences). Data analysis was performed with FlowJo_V10 software (TreeStar, Ashland, OR, USA).

### 2.3. HLA-E Genotyping

DNA was isolated from PBMCs using QIAamp DNA Blood Mini Kit (Qiagen Iberia, Madrid, Spain). HLA-E genotyping was performed by qPCR to differentiate between HLA-E*0101 and HLA-E*0103. The following in-house primers and LNA probes (Integrated DNA Technologies, Leuven, Belgium) were used: Primer_forward: 5′-GCAGTGGATGCATGGCT-3′; Primer_reverse: 5′-GGTCCTCATTCAGGGTGAGATA-3′; Probe_A-FAM (HLA-E*0101 allele): 5′-CGC + C+T + GTC + GG-3′; Probe_G-YAK (HLA-E*0103 allele): 5′-CGC + C+C + GTCGG-3′. Analysis was performed using StepOne Real-Time PCR System (Thermo Fisher Scientific, Waltham, MA, USA).

### 2.4. KIR Haplotyping

KIR genotyping was performed using LinkSeq KIR Typing kit (One Lambda, Thermo Fisher Scientific), which allows testing all 15 human KIR genes and 2 pseudogenes by StepOne Real-Time PCR System (Thermo Fisher Scientific). Data were exported to SureTyper software (One Lambda, Thermo Fisher Scientific) for identification of KIR genotypes. KIR haplotypes AA and BX were assigned according to Allele Frequency Net Database [37]. B haplotypes carry one or more of the following genes: KIR2DL2, -2DL5, -3DS1, -2DS1, -2DS2, -2DS3 and -2DS5; whereas A haplotypes do not carry any of these genes.

### 2.5. Random Forest Algorithm

A Random Forest algorithm [38] was applied to predict the relapse of patients with CML who meet the criteria for safe treatment interruption and evaluate the resulting accuracy. The selection of these parameters was performed according to the existence of significant differences (*p* < 0.05) in the expression patterns when both groups were compared, and they were as follows: related to NK and NKT cells: CD56+, CD3 ± CD56 + CD16 + IFNγ+, CD3 ± CD56 + CD16 + TNFα+, CD3 ± CD56 + CD16 + GZB +, CD56 + NKG2A +, HLA-E*0101, HLA-E*0103, HLA-E*0101/HLA-E*0101, HLA-E*0103/HLA-E*0103, HLA-E*0101/HLA-E*0103, KIR2DS3, KIR2DL5, KIR haplotype AA, KIR haplotype Bx; related to TCRγδ+ T cells: CD8 ± TCRγδ +. Data from 27 patients on TFR who did not relapse and 15 patients who relapsed within 8 months after treatment discontinuation were included in the analysis. In order to avoid bias in the selection of training, testing and validation sets due to the limited number of samples, we performed a combined feature selection and classification procedure using a Random Forest classifier with a nested K-fold cross validation procedure for each competing algorithm, as previously described [39,40,41,42]. In order to ensure that every fold contains the exact number of samples, the inner loop, which was used to fine tune the hyperparameters of the Random Forest model, applied 5-fold cross validation, whereas the outer loop, which was used to test the error of the best model obtained, applied 6-fold cross validation. The variable importance measure (VIM) allowed the categorization of patients according to the normalized total reduction in the criterion brought by each feature, as determined by Gini importance method [43]. The features that show the highest importance were used to classify polymorphisms regarding their ability to predict the investigated phenotype, which in this case is the highest likelihood of relapse.

### 2.6. Statistical Analysis

Statistical analysis was performed with Graph Pad Prism 8.0 (Graph Pad Software Inc., San Diego, CA, USA) using ordinary one-way ANOVA and Tukey’s multiple comparisons test, with a single pooled variance. P values < 0.05 were considered statistically significant and were represented as *, **, ***, or **** for *p* < 0.05, *p* < 0.01, *p* < 0.001, or *p* < 0.0001, respectively.

## 3. Results

### 3.1. Patients’ Characteristics

Ninety-three patients diagnosed with chronic phase CML were recruited for this observational, cross-sectional study. Forty-five CML patients were on treatment with TKIs (henceforth, On TKI; dasatinib *n* = 18 (40%); imatinib *n* = 11 (24.4%); nilotinib *n* = 9 (20%); bosutinib *n* = 4 (8.9%); ponatinib *n* = 1 (2.3%); asciminib *n* = 2 (4.4%)). Median time of treatment was 37.2 months (interquartile range (IQR) 12 to 70.5). Twenty-seven CML patients were on sustained TFR (henceforth, Off TKI). Median time of treatment until controlled interruption was 52.2 months (IQR 40.5 to 102.9). Median time on TFR until the collection of blood samples was 21.6 months (IQR 9.6 to 28.8). The last TKI before discontinuation was nilotinib (*n* = 13; 48.1%), imatinib (*n* = 9; 33.3%), or dasatinib (*n* = 5; 18.5%). Blood samples were also collected from 15 patients who had lost DMR during TFR (henceforth, Relapsed). Median time of treatment until controlled interruption was 55.2 months (IQR 42.6 to 74.1). Median time to relapse after treatment interruption was 4.8 months (IQR 3.6 to 7.5). The last TKI before discontinuation was dasatinib (*n* = 5; 33.3%), nilotinib (*n* = 4; 26.7%), imatinib (*n* = 4; 26.7%), or bosutinib (*n* = 2; 13.3%). Six patients recently diagnosed with CML were also recruited and blood samples were taken before starting TKI treatment (henceforth, new CML diagnosis). A similar proportion of males (50; 53.8%) and females (43; 46.2%) was considered. Median age of patients Off TKI on sustained TFR was 48.5 years (IQR 39.7 to 65), whereas patients Off TKI who relapsed were younger (median age was 38 years, IQR 27 to 57). Prognostic Sokal risk was low in 67 patients (72%) and sixty-five patients (69.9%) showed DMR. Table 2 shows detailed clinical characteristics of all patients.

### 3.2. Profile and Cytokine Production of NK Cell Populations Induced by Treatment with TKIs

**Figure 1:**
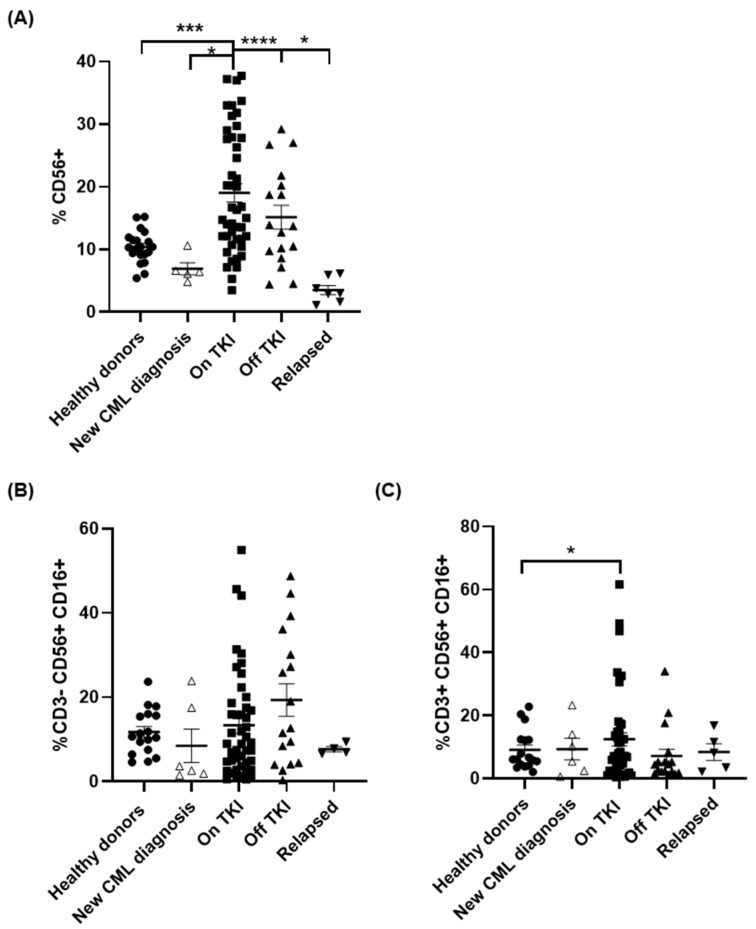
Decrease in Natural Killer (NK) cell populations in peripheral blood of patients with CML who relapsed after treatment interruption. Analysis by flow cytometry of the expression of total CD56+ cells (**A**), NK cells with profile CD3-CD56 + CD16+ (**B**) and NKT cells with profile CD3 + CD56 + CD16+ (**C**) in peripheral blood from the different groups of patients and healthy donors (Healthy donors (●), new CML diagnosis (△), patients On TKI (■), patients Off TKI who did not relapse (▲), patients Off TKI who relapsed (▼)). Each dot corresponds to one sample and lines represent mean ± standard error of the mean (SEM). Statistical significance was calculated using one-way ANOVA and Tukey’s multiple comparisons test. * *p* < 0.05; *** *p* < 0.001; **** *p* < 0.0001.

Expression of CD56 was increased 1.8-fold in patients On TKI in comparison with healthy controls (*p* < 0.01), whereas it was reduced 1.5- and 2.8-fold (*p* < 0.05) in patients with new CML diagnosis in comparison with healthy donors and patients On TKI, respectively (Figure 1A). This high expression of CD56 was maintained, although to slightly lower levels, in patients Off TKI with sustained DMR for more than 2 years. However, CD56 was reduced 4.3-fold in patients who relapsed (*p* < 0.05). The expression of CD16 was analyzed in CD56+ cells in both CD3- and CD3+ populations in order to differentiate between NK (Figure 1B) and NKT (Figure 1C) cells, respectively. There were no significant differences among groups but interestingly, although CD3-CD56 + CD16+ cell population was increased in patients Off TKI, it was reduced to basal levels in patients who relapsed (Figure 1B).

**Figure 2:**
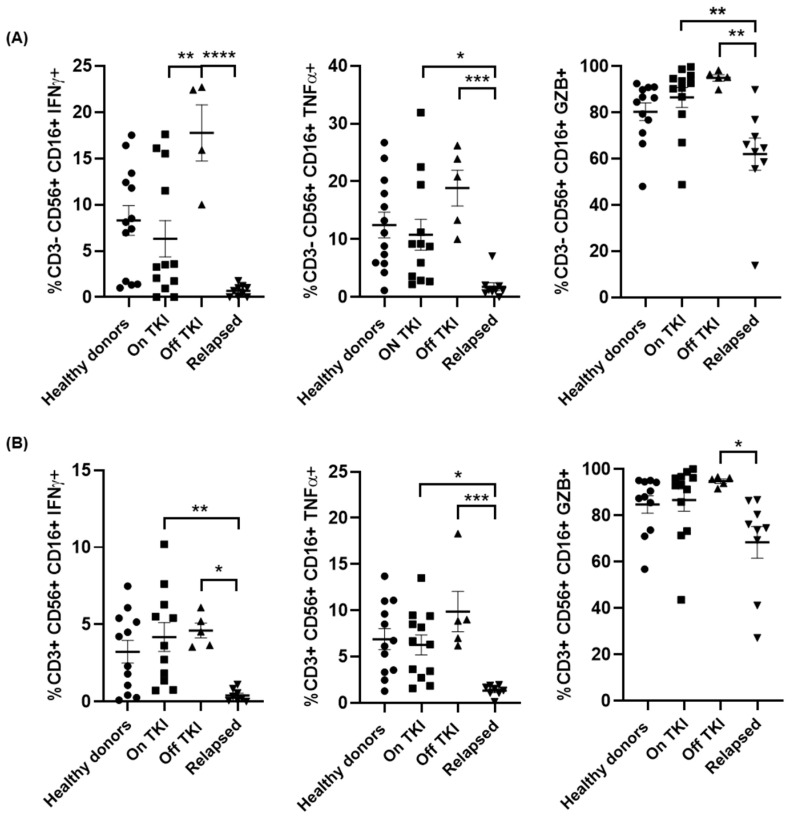
Synthesis of cytokines from NK and NKT cells from PBMCs of different groups of patients with CML. Analysis by flow cytometry of the intracellular expression of interferon gamma (IFNγ), tumor necrosis factor alfa (TNFα) and granzyme B (GZB) in CD3- CD56+ CD16+ NK cells (**A**) and CD3+ CD56+ CD16+ NKT cells (**B**) from each group of patients and healthy donors (Healthy donors (●), patients On TKI (■), patients Off TKI who did not relapse (▲), and patients Off TKI who relapsed (▼)). Each dot corresponds to one sample and lines represent mean ± SEM. Statistical significance was calculated using one-way ANOVA and Tukey’s multiple comparisons test. * *p* < 0.05; ** *p* < 0.01; *** *p* < 0.001; **** *p* < 0.0001.

We also evaluated the functionality of NK and NKT cell populations by measuring the synthesis of IFNγ, TNFα and GZB in response to Hsp70 peptide [44]. Patients Off TKI had CD3-CD56 + CD16+ NK cells in peripheral blood with high capacity to synthesize IFNγ and TNFα, which were increased 2.8-fold (*p* < 0.01) and 1.7-fold, respectively, in comparison with patients On TKI (Figure 2A, left and middle graphs). However, the capacity to synthesize IFNγ and TNFα of CD3-CD56 + CD16+ NK cells from patients who relapsed was reduced 34-fold (*p* < 0.01) and 18-fold (*p* < 0.01), respectively, in comparison with patients Off TKI. Similar results were observed for the induction of the synthesis of GZB from NK cells, which was reduced 1.5-fold (*p* < 0.01) in patients who relapsed in comparison with patients Off TKI (Figure 2A, right graph). In CD3 + CD56 + CD16+ NKT cells, the synthesis of IFNγ and TNFα was reduced 11.6- (*p* < 0.01) and 7.4-fold (*p* < 0.05), respectively, in patients who relapsed in comparison with patients Off TKI (Figure 2B, left and middle graphs). The synthesis of GZB from NKT cells after stimulation was similar to NK cells, as it was reduced 1.4-fold (*p* < 0.05) in patients who relapsed in comparison with patients Off TKI (Figure 2B, right graph).

### 3.3. Differential Expression of Stimulatory and Inhibitory NK Markers

**Figure 3:**
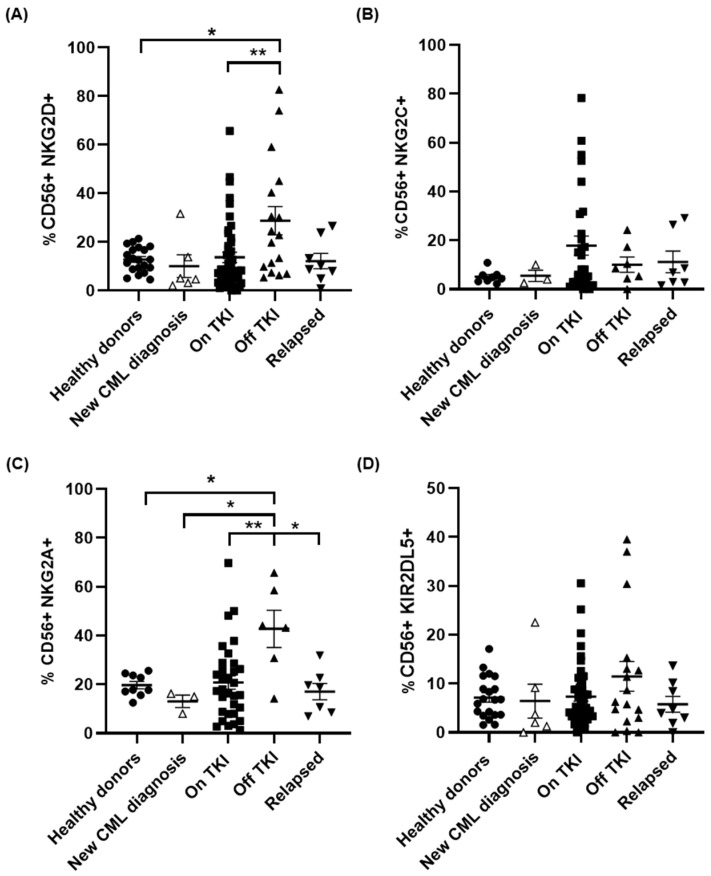
Differential expression of activating or inhibitory receptors in NK cells in the different groups of patients. Analysis by flow cytometry of the expression of the NK activating markers NKG2D (**A**) and NKG2C (**B**) and the NK inhibitory markers NKG2A (**C**) and KIR2DL5 (**D**) on the surface of CD56+ cells in peripheral blood of the different groups of patients and healthy donors (Healthy donors (●), new CML diagnosis (△), patients On TKI (■), patients Off TKI who did not relapse (▲), patients Off TKI who relapsed (▼)). Each dot corresponds to one sample and lines represent mean ± SEM. Statistical significance was calculated using one-way ANOVA and Tukey’s multiple comparisons test. * *p* < 0.05; ** *p* < 0.01.

The expression of NKG2D in CD56+ cells from patients On TKI was similar to that of healthy donors, but it was increased more than 2-fold in patients Off TKI regarding healthy donors (*p* < 0.05) and patients On TKI (*p* < 0.01) (Figure 3A). This expression returned to basal levels in patients who lost DMR during TFR. We did not observe significant differences between groups in the expression of NKG2C. However, NKG2C increased 3.2-fold on the surface of NK cells from patients On TKI, in comparison with healthy donors (Figure 3B), and it decreased 1.7-fold in patients Off TKI who did not relapse. No changes from basal levels were observed in patients who relapsed. The expression of NKG2A increased 2.2-fold in patients Off TKI in comparison with healthy donors (*p* < 0.05) and patients On TKI (*p* < 0.01) (Figure 3C). This expression decreased 2.5-fold in patients who relapsed (*p* < 0.05). The expression of the inhibitory receptor KIR2DL5/CD158f was not significantly modified between groups (Figure 3D), but interestingly it increased 1.6-fold in patients Off TKI and returned to basal levels in CD56+ cells from patients who relapsed.

### 3.4. Treatment with TKIs Increased CD8± TCRγβ+ T Cell Populations

**Figure 4:**
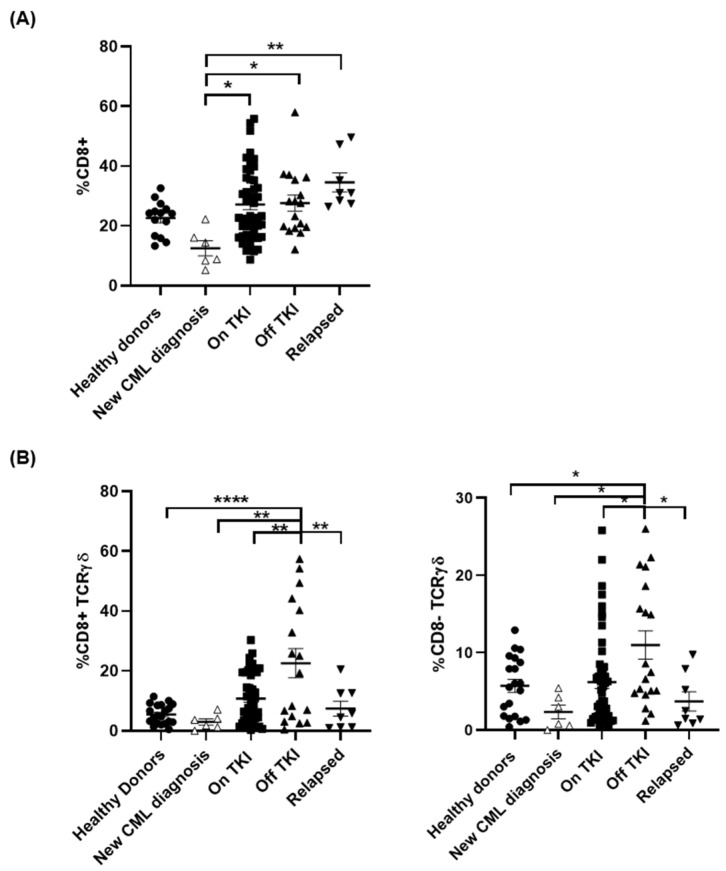
Changes of TCRγβ+ cell populations in peripheral blood from patients with CML who relapsed during treatment interruption. Analysis by flow cytometry of the expression of total CD8+ cells (**A**), as well as CD8 ± T cells expressing TCRγβ (**B**), was performed in peripheral blood of the different groups of patients and healthy donors (Healthy donors (●), new CML diagnosis (△), patients On TKI (■), patients Off TKI who did not relapse (▲), patients Off TKI who relapsed (▼)). Each dot corresponds to one sample and lines represent mean ± SEM. Statistical significance was calculated using one-way ANOVA and Tukey’s multiple comparisons test. * *p* < 0.05; ** *p* < 0.01; **** *p* < 0.0001.

Levels of CD8+ T cells were reduced 1.8-fold in PBMCs from patients with new diagnosis of CML in comparison with healthy donors (Figure 4A). Treatment with TKIs returned CD8+ T cell populations to basal levels (*p* < 0.05) and they were maintained in patients Off TKI (*p* < 0.05). CD8+ T cells slightly increased in patients who relapsed in comparison with patients Off TKI but this increase was not statistically significant. Unconventional CD8+ TCRγβ+ T cells were increased 3.6-fold in patients On TKI in comparison with healthy donors and patients with new diagnosis of CML, still untreated (Figure 4B, left graph). This cell population increased 4.1-fold in patients Off TKI in comparison with healthy donors (*p* < 0.0001) but they were reduced to basal levels in patients who relapsed (*p* < 0.01). The population of CD8- TCRγβ+ cells did not change in patients On TKI, but it increased 1.9-fold in patients Off TKI, in comparison with healthy donors (*p* < 0.05) and patients On TKI (*p* < 0.05) (Figure 4B, right graph). CD8-TCRγβ+ cells returned to basal levels in patients who relapsed (*p* < 0.05).

### 3.5. Predominant HLA-E Homozygosis and KIR Haplotype BX in Patients Who Relapsed during TFR

**Figure 5:**
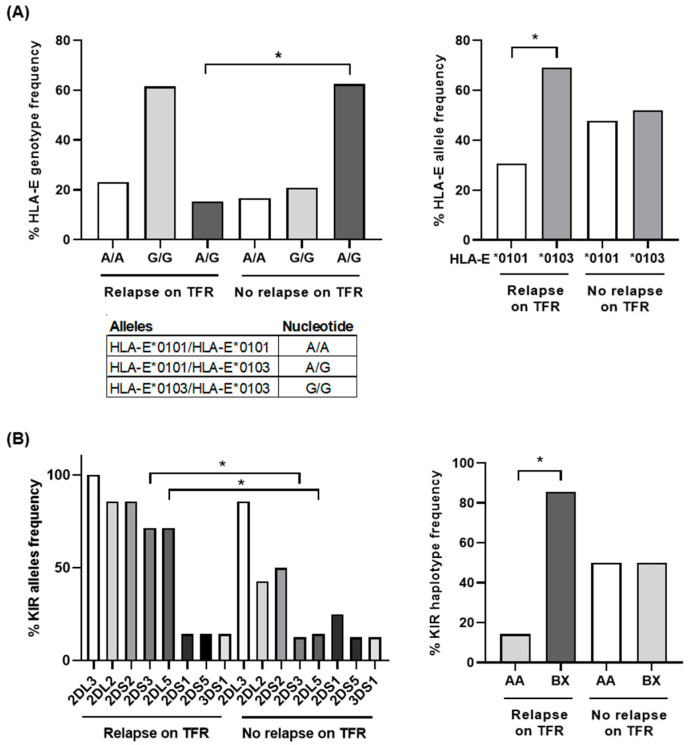
HLA-E and killer cell Ig-like receptor (KIR) genotyping in different groups of patients with CML. (**A**) Analysis by qPCR of the percentage of HLA-E genotyping and alleles frequency in patients with CML who relapsed during treatment interruption (*n* = 13) in comparison with patients with CML who did not relapse (*n* = 24). (**B**) Analysis of the percentage of KIR alleles and haplotypes frequency was performed in patients with CML who relapsed during treatment interruption (*n* = 7) or not (Off TKI) (*n* = 8). Each dot corresponds to one sample and lines represent mean ± SEM. Statistical significance was calculated using one-way ANOVA and Tukey’s multiple comparisons test. * *p* < 0.05.

Most patients (84.6%) who relapsed were homozygous for HLA-E (Figure 5A, left graph), and 76.9% had the HLA-E*0103 allele (*p* < 0.05) (Figure 5A, right graph). Sixty-two percent (62.5%) of patients Off TKI were heterozygous for HLA-E (Figure 5A, left graph) and a similar percentage of HLA-E*0101 (46%) and HLA-E*0103 (54%) frequency was observed within this group (Figure 5A, right graph).

Inhibitory genes KIR2DL2/CD158b and KIR2DL5/CD158f and activating genes KIR2DS2/CD158j and KIR2DS3 were more frequent (86%, 71% (*p* < 0.05), 86%, and 71% (*p* < 0.05), respectively) in patients who relapsed in comparison with patients Off TKI (43%, 14% (*p* < 0.05), 50%, and 13% (*p* < 0.05), respectively) (Figure 5B, left graph). Most patients who relapsed (85.7%) showed KIR BX haplotype (*p* < 0.05), whereas patients who did not relapse (Off TKI) showed a balanced frequency of KIR AA (50%) and BX (50%) haplotypes (Figure 5B, right graph).

### 3.6. Application of Random Forest Algorithm

**Figure 6:**
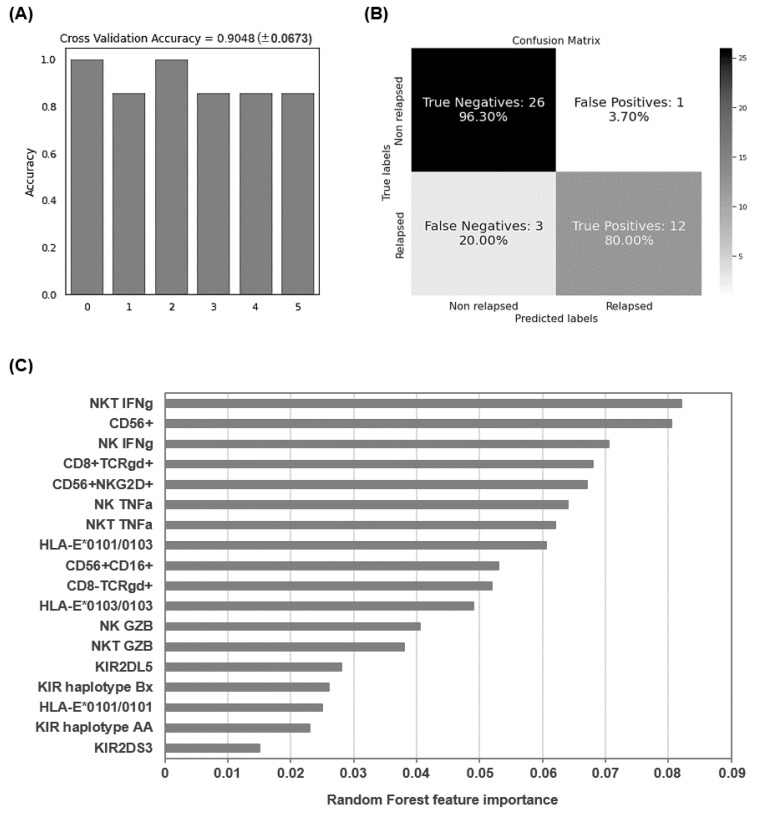
Application of Random Forest algorithm to evaluate the accuracy of the predictive biomarkers of CML relapse. (**A**) Accuracy for the 6 iterations of the outer loop of the nested K-fold cross validation. (**B**) Confusion matrix confronting the true relapse/non-relapse conditions of the patients with CML and the conditions predicted by the algorithm. The Random Forest model predicted 96.3% true negatives and 80.0% true positives, which corresponded to correct predictions of patients who did not relapse and patients who relapsed, respectively. (**C**) The importance of the analyzed features for the categorization of patients with higher likelihood to relapse was analyzed by Gini VIM method.

The Random Forest algorithm was applied to evaluate the accuracy of the analyzed parameters in which there were statistically significant differences in the expression patterns to predict the relapse of patients with CML who interrupted treatment. For this analysis, data of parameters that changed with statistical significance from 27 patients Off TKI and 15 patients who relapsed during TFR were included for this analysis. After the selection of training and testing sets as described above, an accuracy of 90.48 ± 6.73% was obtained for the six iterations of the outer loop of the nested K-fold cross validation for each competing algorithm (Figure 6A). Therefore, 26 out of 27 patients Off TKI (96.30%) and 12 out of 15 patients who relapsed during TFR (80.00%) were correctly assigned to the right group using these features (Figure 6B). The wrong predictions corresponded to three false negatives (prediction that one patient did not relapse when he/she had lost DMR) and one false positive (prediction that one patient had relapsed when he/she had not lost DMR). Proportionally, the non-relapsed group produced more accurate results because more samples were available to perform the training process. Besides, the Gini VIM method determined that variations in immune cell populations such as NK, NKT and CD8+ TCRγβ+ T cells and their ability to release proinflammatory cytokines, as well as HLA-E heterozygosis, were more important than KIR haplotypes for the categorization of patients with higher likelihood to relapse (Figure 6C).

## 4. Discussion

Treatment for life with TKIs was initially recommended for patients with CML in order to avoid relapse in case of discontinuation. However, it is possible to attempt controlled treatment interruption in patients with very low or undetectable levels of BCR/ABL1 for at least two to three consecutive years [45]. Several factors may influence the outcome during TFR but the control of the residual leukemic cells by the immune system is essential [16,19]. Ninety-three patients at the chronic stage of CML, most of them (72%) with low Sokal risk, were recruited to analyze immunological parameters in PBMCs that may be useful as predictive biomarkers of relapse during controlled treatment interruption. Similar percentages of males and females were observed in all groups, although the relationship between gender and CML relapse was not conclusive. Patients who relapsed during TFR were 11 years younger than patients who did not relapse, suggesting that younger age could be a negative factor for maintaining DMR during TFR. However, due to controversial previous reports [46,47,48], current recommendations for the clinical management of CML patients do not consider age an essential factor [49].

TKIs show a direct activity against BCR-ABL+ cells but they are also immunomodulatory drugs that enhance the immune response. Treatment with dasatinib induces the expansion of LGLs, but this event has been associated with a concomitant viral infection such as CMV [16,18,50,51,52,53,54]. This might indicate that further stimulation of the immune system with a chronic viral infection could trigger a sustained response against residual leukemic cells during TFR. In our study, most patients were seropositive for CMV in both groups of relapsed and sustained TFR (Figure S1). Therefore, although the presence of CMV may enhance the antileukemic response, it was not a decisive factor to maintain this response and avoid relapse. On the other hand, the level of NK cells expressing CD56 was greatly increased in CML patients on treatment with TKIs, which is in agreement with previous reports that describe NK cells as the main component of LGLs [17,55]. Moreover, patients on TFR without relapsing maintained these high levels of CD56+ cells, whereas patients who relapsed showed very low levels of these cells. Therefore, the maintenance of a sustained population of NK cells seemed essential for a continuous antileukemic control in the absence of treatment. The level of NK and NKT activation was also different in both groups of patients as the expression of most essential activating NK markers was reduced in cells from patients who relapsed, such as CD16 and some CD94/NKG2 receptors. Although without statistical significance, the expression of NKG2D was reduced in patients who relapsed. The activation of NKG2D induces the reactivity of NK cells against tumor cells [56], but the expression of this marker is negatively modulated in patients with CML and other types of cancer, which facilitates the escape of tumor cells [57]. However, treatment with TKIs modulated NKG2D expression, which would favor the lytic activity of NK cells [25]. We also observed a statistically significant reduction in the expression of NKG2A in patients who relapsed, which conversely has been previously related to better prognosis [24]. The high expression of NKG2C has also been associated to reduced CML relapse [25], but it was not significantly different between patients who relapsed or not during treatment discontinuation. Accordingly, in our study, the expression of several CD94/NKG2 receptors was reduced overall in patients who relapsed, which was in accordance with the decreased functionality of NK cells in blood from these patients. This was demonstrated by the reduced synthesis of proinflammatory cytokines such as IFN or TNFα and cytotoxic proteases such as GZB after the stimulation of both NK and NKT cells from patients who relapsed.

Other cytotoxic cells such as CD8+ T lymphocytes (CTLs) may also participate in the control of leukemic cells during CML, although it was described that they cannot eliminate the cancerous clone completely due to progressive exhaustion [58]. However, the presence of specific CTLs, even at low frequency and with impaired functionality, may be a positive factor towards the control of CML during TFR since complete depletion of CTLs in animal models leads to rapid progression of the disease and death [59]. We did not find significant differences in the expression of the exhaustion marker PD-1 between PBMCs of different groups of patients (Figure S2). However, we observed a significant increase in unconventional CD8± T cells expressing TCRγβ+ in PBMCs from patients on TFR who did not relapse. These patients had not only been on treatment with dasatinib, as described before [50], but also with other TKIs such as imatinib or nilotinib. Conversely, the presence of these cells was greatly reduced in patients who relapsed, independently of the treatment they received. CD8± TCRγβ+ cells have been appointed as a cell-based immunotherapy against cancer [60] due to their potent lytic activity that is not restricted by MHC presentation [29,30]. Therefore, high levels of CD8± TCRγβ+ cells may also contribute to maintaining DMR during TFR.

Low polymorphic HLA-E molecules are ligands of CD94/NKG2, thereby regulating lysis mediated by NK, NKT and TCRγβ+ cells during the cytotoxic response [31,61,62]. In our study, most patients who relapsed (76.9%) were homozygous for HLA-E*0103. This frequency was 1.7-fold higher than that estimated for the general population in Europe (43.3%) [63]. HLA-E*0103 has been associated with higher susceptibility to cancer diseases such as acute leukemia [64] and ovarian cancer [65], as well as with an increased risk of mortality in patients with chronic lymphocytic leukemia [66]. However, no association between cancer and HLA-E*0101 has been reported so far [67]. Both alleles differ only in one amino acid (Arg107Gly) that leads to higher stability of HLA-E*0103 on the cell surface, which increases half-life and prolongs the interaction with immune effector cells [68]. Both alleles present a limited set of peptides derived from class I leader sequences, although HLA-E*0101 may also present non-canonical peptides in the absence of HLA-class I molecules [69]. Therefore, the beneficial role of HLA-E*0101 for avoiding relapse of CML could be related to its decreased expression on the cell surface, which could result in lower inhibitory activity of NKG2A+ cells [70], and to a less restrictive peptide repertoire presentation than that found with HLA-E*0103 [71]. Furthermore, HLA-E*0103 allele has been associated with high levels of soluble HLA-E (sHLA-E), which provides protection against NK-mediated lysis [72] and usually correlates with disorders such as cancer and autoimmune diseases [67]. To our knowledge, this is the first report that describes a possible association between higher susceptibility to CML relapse and HLA-E*0103 homozygosis, which highlights the usefulness of this allele as biomarker.

The activity of NK and TCRγβ+ T cells is also modulated by the KIR repertoire expressed on the cell surface. The role of specific KIR genes as risk factors for leukemia is controversial but the absence of KIR ligands has been associated with lower risk of relapse in patients with CML who received allogeneic HSC transplantation from unrelated donors [26]. In our study, KIR2DS3 was more frequent in patients who relapsed after treatment interruption. This transmembrane glycoprotein mediates NK cytotoxicity and, although its expression is reduced in patients who develop acute leukemia [27], it is an important risk factor for CML relapse [26]. KIR2DL5B was also present in most patients who relapsed during TFR. KIR2DL5 is an NK receptor with two isoforms A and B that inhibits cytotoxicity, preventing the lysis of target cells [73]. The presence of KIR2DL5B is a predictor of inferior molecular response in CML, as well as reduced transformation-free survival and event-free survival [74]. KIR haplotype was determined for the patients included in our study based on KIR genes content. B haplotypes contain one or more B-specific genes such as KIR2DS1, KIR2DS2, KIR2DS3, KIR2DS5, KIR2DL2, and KIR2DL5 [75]. Most patients who relapsed during TFR showed KIR haplotype BX, which may contain inhibitory KIR2DL5B and activating KIR2DS3 genes. According to our results, other studies described previously that BX haplotype is significantly associated with CML relapse [76]. However, although there is still controversy about using KIR haplotyping as a predictive parameter of CML remission outcome [17,77], the importance of this feature as a predictive biomarker was lower than that of the high levels of functional cytotoxic cells and the presence of HLA-E*0103.

Consequently, in this study we defined several parameters related to the antileukemic immune response that showed significantly different expression in PBMCs from patients who relapsed during TFR in comparison with patients on sustained TFR. Some of these parameters were in accordance with previous reports [78] and other were newly described. In order to determine the applicability as predictive biomarkers of CML relapse of the parameters evaluated in this study, we used a Random Forest algorithm. Although there was some class imbalance between the number of relapsed and non-relapsed patients, we overcame this issue by using a nested K-fold cross validation procedure for each competing algorithm. According to the model, the selected parameters could predict the classification of patients with CML in groups of relapse or non-relapse during TFR with 90.48% accuracy.

## 5. Conclusions

Several immune factors should be considered as predictors of CML relapse after treatment interruption such as low levels of CD56+ cells with low expression of CD16 and CD94/NKG2 receptors, impaired synthesis of proinflammatory cytokines or cytotoxic proteases from NK and NKT cells, low levels of CD8± TCRγβ+ T cells, as well as KIR haplotype BX and homozygosis for HLA-E*0103, which is a potential predictive biomarker never explored before. According to our results, the development of functional cell populations with cytotoxic potential in patients on successful TFR was not related to treatment with a particular TKI or to a previous infection with CMV but it seemed more dependent on the ability of the immune system of each patient to answer continuously against the residual leukemic cells. Applied to clinical practice, these biomarkers will help to decide which patients with CML may safely initiate a controlled treatment interruption with a high likelihood of non-relapse. The applicability of these biomarkers will be tested in a longitudinal study with a larger cohort of patients.

## Figures and Tables

**Table 1:** Summary of clinical data of patients with chronic myeloid leukemia (CML) that participated in the study.

		New CML Diagnosis	CML on TKI	CML off TKI	CML Relapsed
**Patients (*n*)**		6	45	27	15
**Male/female (*n*)**		3/3	22/23	18/9	7/8
**Median age (years)**		54.5y (IQR 44.7 to 64)	49.0y (IQR 37.5 to 59)	48.5y (IQR 39.7 to 65)	38.0y (IQR 27 to 57)
Sokal Risk Score (L/I/H/UD) * (*n*)		3/2/1/0	34/6/5/0	17/7/0/3	13/2/0/0
Response at sampling ** (*n*)	**CCyR**	N/A	4	0	10
	**MMR**	N/A	8	0	0
	**DMR**	N/A	33	27	5
	**UD**	N/A	0	0	0

* Sokal Risk, L = low/I = intermediate/H = high/UD = undetermined. ** Response at sampling: CCyR = complete cytogenetic response; MMR = major molecular response; DMR = deep molecular response, IQR = interquartile range; N/A = not applicable; y = years.

**Table 2:** Relevant clinical data of the patients with CML recruited for this study.

	Patient’ Code	Gender (M/F)	Age at Diagnosis (Years)	Sokal Risk	CML Phase: Chronic Accelerated Blast	Hematological Remission (Y/N)	Cytogenetic Remission (Y/N)	Molecular Response (Log)	Last TKI	Time on Treatment with Last TKI (Months)	Time on TFR (Months)
**New CML diagnosis**	1	F	44	Low	Chronic	N/A	N/A	N/A	-	-	-
	2	M	56	High	Chronic	N/A	N/A	N/A	-	-	-
	3	F	45	Low	Chronic	N/A	N/A	N/A	-	-	-
	4	M	57	Intermediate	Chronic	N/A	N/A	N/A	-	-	-
	5	M	85	Intermediate	Chronic	N/A	N/A	N/A	-	-	-
	6	F	53	Low	Chronic	N/A	N/A	N/A	-	-	-
**CML On treatment with TKI**	7	M	35	Low	Chronic	Yes	Yes	5	Imatinib	96	-
	8	F	38	Low	Chronic	Yes	Yes	4	Imatinib	14.4	-
	9	F	62	Low	Chronic	Yes	Yes	4	Imatinib	24	-
	10	F	15	Low	Chronic	Yes	Yes	4.5	Imatinib	12	-
	11	M	52	Low	Chronic	Yes	Yes	5	Imatinib	168	-
	12	F	56	Low	Chronic	Yes	Yes	5	Imatinib	156	-
	13	F	52	Intermediate	Chronic	Yes	Yes	5	Imatinib	32.4	-
	14	M	75	Intermediate	Chronic	Yes	Yes	3	Imatinib	6	-
	15	M	65	Low	Chronic	Yes	Yes	5	Imatinib	15.6	-
	16	M	38	Low	Chronic	Yes	Yes	5	Imatinib	132	-
	17	M	34	Low	Chronic	Yes	Yes	5	Imatinib	40.8	-
	18	F	49	Low	Chronic	Yes	Yes	4	Dasatinib	30	-
	19	F	27	Low	Chronic	Yes	Yes	4.5	Dasatinib	9.6	-
	20	M	55	Low	Chronic	Yes	Yes	3	Dasatinib	30	-
	21	F	41	Low	Chronic	Yes	Yes	No	Dasatinib	1.2	-
	22	M	24	Low	Chronic	Yes	Yes	3	Dasatinib	84	-
	23	F	57	Intermediate	Chronic	Yes	Yes	3	Dasatinib	34.8	-
	24	F	59	Low	Chronic	Yes	Yes	4	Dasatinib	30	-
	25	F	57	Low	Chronic	Yes	Yes	3	Dasatinib	72	-
	26	M	39	High	Chronic	Yes	Yes	No	Dasatinib	1.2	-
	27	F	56	High	Chronic	Yes	Yes	5	Dasatinib	48	-
	28	F	55	High	Chronic	Yes	Yes	5	Dasatinib	4.8	-
	29	M	43	Low	Chronic	Yes	Yes	4.5	Dasatinib	12	-
	30	M	44	Intermediate	Chronic	Yes	Yes	4.5	Dasatinib	84	-
	31	F	60	Intermediate	Chronic	Yes	Yes	5	Dasatinib	12	-
	32	F	43	Low	Chronic	Yes	Yes	5	Dasatinib	42	-
	33	F	59	Intermediate	Chronic	Yes	Yes	5	Dasatinib	12	-
	34	M	72	Low	Chronic	Yes	Yes	4	Dasatinib	48	-
	35	M	26	Low	Chronic	Yes	Yes	5	Dasatinib	80.4	-
	36	F	35	Low	Chronic	Yes	Yes	4.5	Nilotinib	48	-
	37	M	35	Low	Chronic	Yes	Yes	5	Nilotinib	63.6	-
	38	F	43	Low	Chronic	Yes	Yes	No	Nilotinib	72	-
	39	F	59	High	Chronic	Yes	Yes	4	Nilotinib	30	-
	40	M	41	Low	Chronic	Yes	Yes	4	Nilotinib	79.2	-
	41	M	37	Low	Chronic	Yes	Yes	5	Nilotinib	60	-
	42	F	67	High	Chronic	Yes	Yes	5	Nilotinib	72	-
	43	M	47	Low	Chronic	Yes	Yes	5	Nilotinib	66	-
	44	M	49	Low	Chronic	Yes	Yes	5	Nilotinib	39.6	-
	45	M	72	Low	Chronic	Yes	Yes	4	Bosutinib	54	-
	46	F	54	Low	Chronic	Yes	Yes	4	Bosutinib	9.6	-
	47	M	29	Low	Chronic	Yes	Yes	3	Bosutinib	39.6	-
	48	F	62	Low	Chronic	Yes	Yes	3	Bosutinib	26.4	-
	49	M	29	Low	Chronic	Yes	Yes	No	Ponatinib	UD	-
	50	F	47	Low	Chornic	Yes	Yes	4	Asciminib	9.6	-
	51	M	60	Low	Chronic	Yes	Yes	3	Asciminib	1.2	-
**Treatment withdrawal without CML relapse**	52	M	39	Intermediate	Chronic	Yes	Yes	4	Imatinib	20.4	12
	53	M	49	Low	Chronic	Yes	Yes	4	Imatinib	180	13.2
	54	F	38	Low	Chronic	Yes	Yes	5	Imatinib	171.6	16.8
	55	F	55	Low	Chronic	Yes	Yes	5	Imatinib	168	7.2
	56	M	41	Low	Chronic	Yes	Yes	5	Imatinib	178.8	24
	57	F	42	Low	Chronic	Yes	Yes	5	Imatinib	182.4	24
	58	F	61	Low	Chronic	Yes	Yes	4	Imatinib	156	28.8
	59	M	71	Low	Chronic	Yes	Yes	5	Imatinib	39.6	7.2
	60	M	34	Low	Chronic	Yes	Yes	5	Imatinib	40.8	8.4
	61	M	46	Intermediate	Chronic	Yes	Yes	5	Dasatinib	54	13.2
	62	M	26	Low	Chronic	Yes	Yes	5	Dasatinib	60	21.6
	63	M	31	Intermediate	Chronic	Yes	Yes	5	Dasatinib	85.2	58.8
	64	M	U/D	UD	Chronic	Yes	Yes	4–5	Dasatinib	UD	24
	65	M	72	Low	Chronic	Yes	Yes	4	Dasatinib	48	8.4
	66	M	61	Low	Chronic	Yes	Yes	4	Nilotinib	52.8	54
	67	F	48	Low	Chronic	Yes	Yes	5	Nilotinib	15.6	15.6
	68	F	71	Intermediate	Chronic	Yes	Yes	5	Nilotinib	51.6	44.4
	69	M	74	Intermediate	Chronic	Yes	Yes	5	Nilotinib	39.6	40.8
	70	M	53	Low	Chronic	Yes	Yes	4.5	Nilotinib	43.2	42
	71	F	33	Low	Chronic	Yes	Yes	5	Nilotinib	44.4	40.8
	72	F	83	UD	Chronic	Yes	Yes	4–5	Nilotinib	24	6
	73	M	61	UD	Chronic	Yes	Yes	4–5	Nilotinib	24	6
	74	M	40	Low	Chronic	Yes	Yes	4–5	Nilotinib	60	12
	75	F	63	Intermediate	Chronic	Yes	Yes	4–5	Nilotinib	44.4	26.4
	76	M	74	Intermediate	Chronic	Yes	Yes	4–5	Nilotinib	44.4	26.4
	77	M	41	Low	Chronic	Yes	Yes	5	Nilotinib	79.2	9.6
	78	M	47	Low	Chronic	Yes	Yes	5	Nilotinib	66	27.6
**CML relapsed**	79	F	61	Intermediate	Chronic	Yes	Yes	No	Imatinib	40.8	14.4
	80	M	35	Low	Chronic	Yes	Yes	No	Imatinib	39.6	4.8
	81	M	71	Low	Chronic	Yes	Yes	No	Imatinib	39.6	9.6
	82	F	28	Low	Chronic	Yes	Yes	4	Imatinib	60	8.4
	83	M	48	Low	Chronic	Yes	Yes	No	Dasatinib	43.2	3.6
	84	M	26	Low	Chronic	Yes	Yes	No	Dasatinib	80.4	3.6
	85	F	25	Low	Chronic	Yes	Yes	4	Dasatinib	60	7.2
	86	F	36	Low	Chronic	Yes	Yes	4.5	Dasatinib	60	4.8
	87	F	49	Low	Chronic	Yes	Yes	4	Dasatinib	180	2.4
	88	M	45	Low	Chronic	Yes	Yes	No	Nilotinib	228	UD
	89	M	22	Low	Chronic	Yes	Yes	No	Nilotinib	50.4	3.6
	90	M	57	Intermediate	Chronic	Yes	Yes	No	Nilotinib	48	6
	91	F	38	Low	Chronic	Yes	Yes	4.5	Nilotinib	72	3.6
	92	F	27	Low	Chronic	Yes	No	No	Bosutinib	48	1.2
	93	F	57	Low	Chronic	No	No	No	Bosutinib	UD	7.2

## Supplementary Materials

The following are available online at https://www.mdpi.com/2077-0383/10/1/42/s1, Figure S1: Titers of IgGs against CMV in plasma from the different groups of patients with CML, Figure S2: Changes in the expression of PD1 in mononuclear cells from peripheral blood of patients with CML.
