# Supplementary material for: Identification of Immunological Parameters as Predictive Biomarkers of Relapse in Patients with Chronic Myeloid Leukemia on Treatment-Free Remission
Source: J Clin Med. 2020 Dec 25;10(1):42. doi: 10.3390/jcm10010042 (PMC7795332; doi:10.3390/jcm10010042)
Supplement: Supplementary file 1 [file jcm-10-00042-s001.pdf]

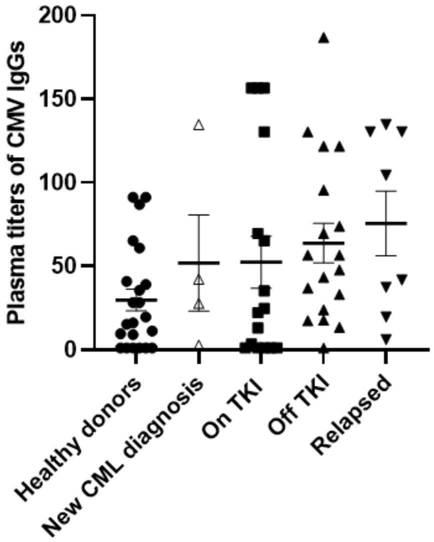

Figure S1. Titers of IgGs against CMV in plasma from the different groups of patients with CML. Plasma samples were analyzed by ELISA to detect specific IgG against CMV using Enzygnost Anti-CMV/IgG kit (Siemens, Marburg, Germany). This test was a single point quantitative assay based in the alpha-method [1], using dilutions 1:231. IgG titer was considered positive when >500; undetermined range was considered between 500 and 231; and <231 was considered negative. All data were normalized according to 231 value as basal. Statistical analysis was performed using one-way ANOVA and Tukey's multiple comparisons test but no significant differences between groups were found. [1] Dopatka HD, Giesendorf B. Single point quantification of antibody by ELISA without need of a reference curve. J Clin Lab Anal 1992, 6:417-422. doi:10.1002/jcla.1860060614.

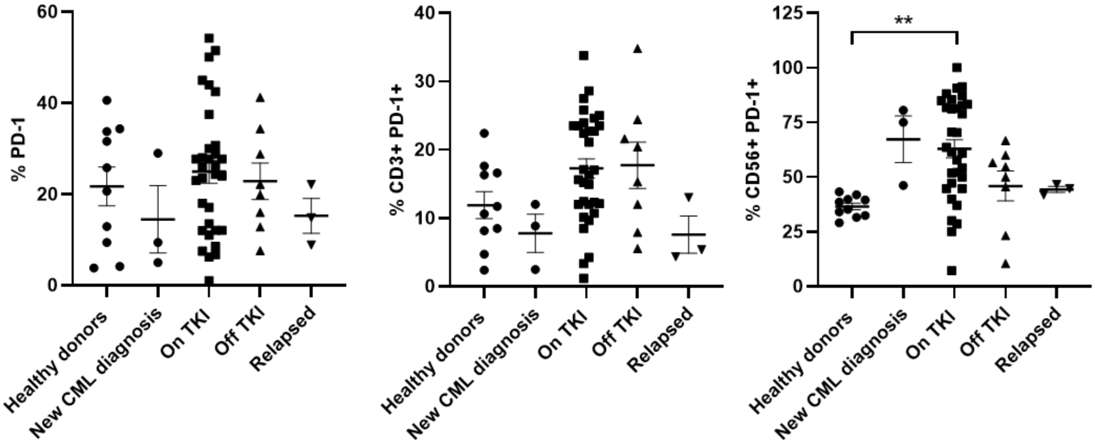

Figure S2. Changes in the expression of PD1 in mononuclear cells from peripheral blood of patients with CML. Levels of total PD1 (a), in CD3+ cells (b) and in CD56+ cells (c) were analyzed by flow cytometry after staining with the corresponding antibodies. PD1-BV650 antibody was purchased from BD Biosciences. Statistical analysis was performed using one-way ANOVA and Tukey's multiple comparisons test. **, $p < 0.01$.
